# Intraductal ultrasonography using an ultrathin radial miniature probe and guide sheath to diagnose a bile duct stricture

**DOI:** 10.1055/a-2607-8228

**Published:** 2025-06-26

**Authors:** Tsuyoshi Suda, Kiichiro Kaji, Miyabi Miura, Kuniaki Arai, Shuichi Terasaki

**Affiliations:** 1 Department of Gastroenterology, Kanazawa Red Cross Hospital, Kanazawa, Japan

A 91-year-old man presented with liver dysfunction: aspartate aminotransferase 81 U/L, alanine aminotransferase 166 U/L, alkaline phosphatase 323 U/L, gamma-glutamyl transferase 527 U/L, total bilirubin 1.6 mg/dL, prothrombin time-international normalized ratio 1.02, and albumin 3.5 g/dL. Magnetic resonance cholangiopancreatography and endoscopic retrograde cholangiopancreatography (ERCP) revealed a distal bile duct stricture ( Fig. 1 ). Laboratory tests revealed an elevated C-reactive protein level (4.30 mg/dL), and acute cholangitis was suspected. After endoscopic biliary drainage had been performed, adenocarcinoma was identified from bile cytology. Contrast-enhanced computed tomography failed to clearly delineate the tumor.

**Fig. 1:**
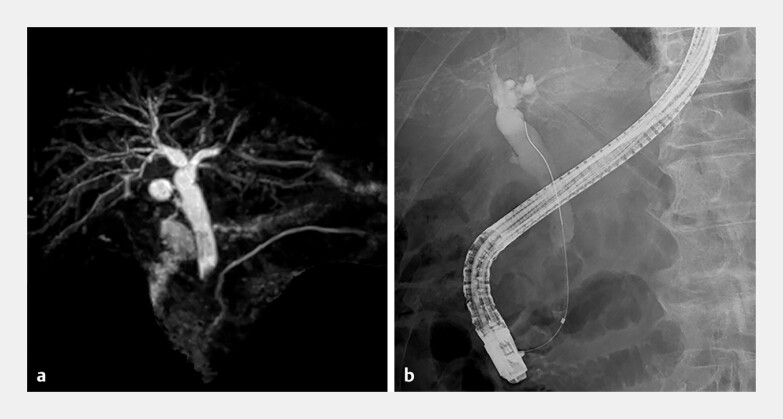
A lower bile duct stricture is shown on: **a** magnetic resonance cholangiopancreatography: **b** endoscopic retrograde cholangiopancreatography.

Further diagnostic evaluation was conducted using ERCP and intraductal ultrasonography (IDUS). An ultrathin radial miniature probe (UM-S20-17S; Olympus, Tokyo, Japan) with a 1.4-mm distal diameter was deployed through a guide sheath system (UMIDAS sheath cannula; UMIDAS, Kanagawa, Japan) ( Fig. 2 ; Video 1 ), as confirmed on fluoroscopic images ( Fig. 3 ). Detailed IDUS at 20 MHz, performed while infusing saline via the guide sheath to distend the bile duct, showed normal proximal bile ducts ( Fig. 4 **a** ), the tumor’s upper margin ( Fig. 4 **b** ), the stricture site ( Fig. 4 **c** ), and serosal layer disruption ( Fig. 4 **d** ). After the IDUS, forceps biopsies were obtained through the guide sheath, which confirmed the diagnosis of adenocarcinoma. The patient opted for best supportive care.

**Fig. 2:**
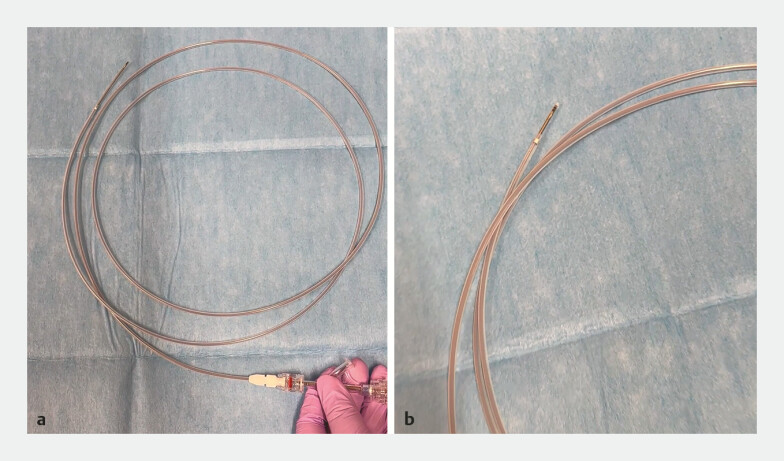
**a, b** Photographs of the ultrathin radial miniature probe being passed through a guide sheath system.

**Fig. 3:**
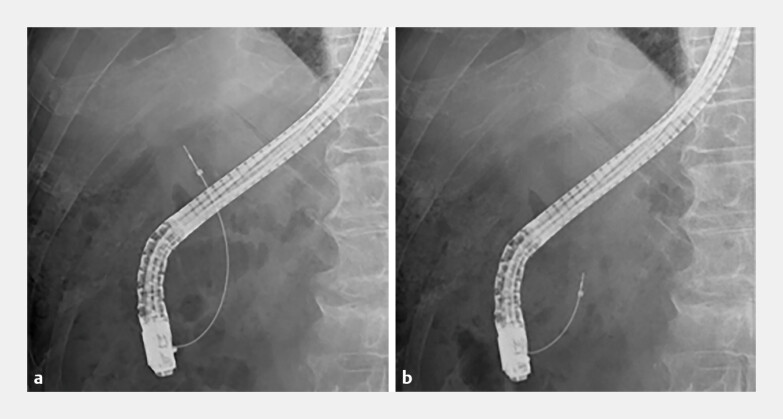
**a, b** Fluoroscopic images showing the ultrathin radial miniature probe passing through the guide sheath system into the bile duct.

**Fig. 4:**
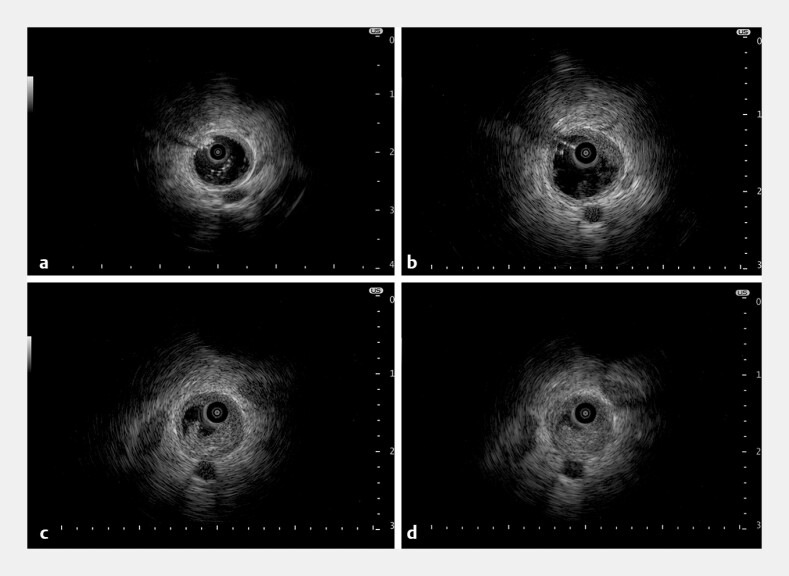
Detailed intraductal ultrasonography images showing: **a** normal proximal bile ducts; **b** the upper margin of the tumor; **c** the stricture site; **d** disruption of the serosal layer.

Ultrasonographic observation of the bile duct using an ultrathin radial miniature probe passed through a guide sheath, performed while infusing saline solution.Video 1

Radial miniature probes, such as the UM-S20-17S, are primarily used in respiratory medicine for endobronchial ultrasound 1 . Despite its potential advantages, the extreme thinness of the UM-S20-17S may explain its limited use during ERCP at many facilities. The guide sheath system was originally developed for selective pancreatic and biliary duct biopsy 2 , but other applications have been reported, including those previously documented by our group 3 4 . IDUS uses a high frequency ultrasonography probe to obtain real-time high quality cross-sectional images during ERCP; however, the challenge of maintaining the probe at the center of the bile duct and the presence of air within the duct can affect image quality, making it difficult to produce clear images 5 . Observing the bile duct using an ultrathin radial miniature probe passes through a guide sheath may address these issues.

Endoscopy_UCTN_Code_TTT_1AR_2AD

## Notes

Endoscopy E-Videos https://eref.thieme.de/e-videos
*E-Videos* is an open access online section of the journal *Endoscopy* , reporting on interesting cases and new techniques in gastroenterological endoscopy. All papers include a high-quality video and are published with a Creative Commons CC-BY license. Endoscopy E-Videos qualify for HINARI discounts and waivers and eligibility is automatically checked during the submission process. We grant 100% waivers to articles whose corresponding authors are based in Group A countries and 50% waivers to those who are based in Group B countries as classified by Research4Life (see: https://www.research4life.org/access/eligibility/ ). This section has its own submission website at https://mc.manuscriptcentral.com/e-videos .
